# Address by Dr. Kate C. Moody

**Published:** 1887-08

**Authors:** Kate C. Moody


### Address.

(Delivered before the Alumni of the Dental College of the University of Michigan, June 29, 1887.)

BY DR. KATE C. MOODY, D.D.S.

It has come to be quite well understood that the time honored custom of an address at the close of college, and on other great occasions is kept up, not so much for any good that is expected to come to those in attendance, as that it gives an opportunity to some favored one to air his pet ideas before an audience which is usually in an affable mood, due to the pleasant surroundings, the general feeling of good-fellowship, and perhaps not a little to a feeling of relief from lectures and "quizzes," and, above all, the dreaded "*finals.*" Under such felicitous circumstances I feel highly honored to be counted among the favored few on an occasion which probably will come but once in any of our lives, for few of us expect to be here when another fifty years have passed away.

When we consider that it is only twelve years since the Dental Department was grafted on to the family tree, the first thought is, that there is not enough about which to make much of a spread. Some of you will not need to go very far back to recall the feelings at the age of twelve. There is not much to look back upon; all was in the future. With dolls and tops and chewing gum, were left behind childish hopes and grievances. You began to think "What will I do when I am grown?" Some older person was propably chosen as an ideal pattern, but, were the Dental Department of the University of Michigan to be regarded as a young miss of twelve, the simile would not be perfect, for she has no call to look to *any* older institution of the kind, either in this or any other country for a *pattern*, even at the age of *twelve*. We say this with due respect to all other colleges as well as to the proverb—"Let another man praise thee; and not thine own mouth," for we think it is with pardonable pride that we can say that our Alma Mater stands in the front line. As one of the younger children, our department likes to watch the older girls (and ape them too, perhaps). She dreams of how *her* children will be filling places of trust and honor in the future, as those of the older ones in the family are now doing.

Would you think it only "such stuff" as dreams are made of should her visions be of a professorship in some college, an elegant office in some European city with professional services in demand among the royalty? or, equally as good, of a skillful, well informed, fair and square dentist in some live town in our own land—"A man among men," respected and honored.

I believe you will all agree with me when I say that it remains with *us* to decide whether these fond, individual hopes shall be realized, or shall end in disappointment. This is an occasion when we can afford to be charitable, so we will take it for granted that we all have made the best of our time while in college; and, we like to think that, could all the children belonging to the Dental branch of this great family be marshalled into line, the fatherly heart of our Dean would beat proudly as he looked upon the throng—or, would there be a sigh of regret as some of us filed by, that his kind heart had overruled his wise head and had refused the deserved "plucking?"

As alumni of this college we are none of us so old or so firmly fixed in our habits that a little good, wholesome advice will come amiss, and especially to the class just graduating would a few words as to the future be allowable, notwithstaning the fact that the younger children object to advice from the older ones. I know just how you all feel, now that the goal is reached and the coveted prize has been won. Oh beautiful season of innocence! when we fondly imagine that the dental lore that is *not* contained in our own weighty notebooks, and no less weighty heads, would make a vest-pocket edition.

It may seem cruel, just in the height of this blissful state to suggest that there will come a time of awakening, when the feeling of depression caused by a failure will refuse to move off to the tune—"It's the way they have at Ann Arbor." The stern realities will stare you in the face till you would give five of the best years of your life just to slip into the faculty room of the Dental College and have a confidential talk with its presiding genius. Then you will begin to realize how much is contaned in the short phrase, "Know thyself," but there are many bright and promising things to offset these dark pictures.

As professional men and women you will be looked upon as the embodiment of science by the laity, if you give them half a chance, and there is no reason why we should not accept and retain this position among our fellowmen, if we would keep our wits about us, our faculties at their best by reading and research, and our minds and hearts pure.

The world has a right to expect something from us. The mental capacity with which we have been endowed, the time and money expended to fit us for our position, were all placed at our disposal by a Higher Power for some higher purpose than the serving of our own advancement alone. Every one should consider himself responsible for the elevating of his own social circle; and the greater the endowment the greater the circle. We should not be content with environments whose limits are our own office, or even our own town. The association with those in our profession has a very *broadening* effect. To some extent such opportunities lie within the reach of all of us. 'Tis true we can not all attend commencement at Ann Arbor every summer, nor can we even go to the American Dental Association each year, but our own State or district will furnish us such opportunity to meet the best men in the profession in our own reach, and I think there are plenty of practitioners who will tell you that they have received an incentive while attending a meeting of their State Society which sent them home with the resolve to cut loose from amalgam and cotton root-fillings, as well as many other good ideas of reform. No matter how good the curriculum of a college, it is impossible for us to gain there the practical finish that we get by mingling with others who are working and thinking in the same line with us. Nor is this all that we need. No matter how full our notes, or how well we have listened to the excellent lectures given us, we will soon find that these are but indices pointing in the right direction, with many an untrodden field of investigation and research lying invitingly beyond.

Experience is indispensable, but along with it, " books are a part of man's prerogative. In formal ink they thought and voices hold."

With a small outlay, there is no reason why such names as Koch, Miller, Foster, Boedecker, Black, and Abbott should not be as familiar to us as our next door neighbors. 'Tis true, it will take a little closer application to become familiar with their theories than it does to read the account of the last base ball game, or the latest fashion magazine. But if there is a real desire for getting at the bottom of these theories, to prove them by personal investigation, not content to take another's word for it, we will be willing to spend the last dollar for a microscope, and leisure time will be very likely to be spent in making sections, staining and mounting specimens, etc., instead of lounging around counting up how much we have lost because that tardy patient kept us waiting.

By making the best use of leisure time, there will be opportunity to further carry out the work of which we had a mere suggestion in the chemical and physiological laboratories, work which we scarcely appreciated while doing it, simply because we did not then realize the value the knowlege would be to us afterwards. Some of us have wished that the course in these departments of work had been much longer, or, better still, that we could return after a few years practice and repeat the work. If the beginner, who will have more spare time than is desirable, would form the habit of thus utilizing it, grand results would follow in the future. At a time when our most advanced thinkers are giving the results of their study to the world in our journals, some acquaintance with their subjects is absolutely necessary, unless we are willing to be left behind.

We do not propose that we all shall drop plugger and mallet and proceed to make *specialists* of ourselves. This would not be practicable. But it lies within the power of any one who has passed the entrance examination, and has done even fair work during the course, to have an intelligent understanding of micro-organisms, femults, etc., sufficient to read intelligently, and have the ability to decide whether a theory is worthy of investigation.

or not. It may seem like deep water at first, but if we verify their statements by our own experiments, it will soon become very fascinating, and we cannot remain uninterested if we would. I know that some will raise the objection of neglect of business by one who is so absorbed in scientific study. While this is *possible*, it is not at all necessary. If it is not possible to do such work without becoming so absorbed that we will keep a patient waiting while we pursue it, then *let it alone*, for we *must* have our bread and butter, and, to obtain it, *must attend to business*, but if our microscope stands ready, it will not take long to remove a little of the accumulation about the necks of the teeth and put it on a slide for examination after the patient leaves, or while waiting for an oxyphosphate filling to harden. The revelation of the number and variety of micro-organisms thus obtained will well repay the trouble, and it certainly is well for us to know something of the enemy with which we have to do battle in our effort to prevent or retard disease. And the free lecture, we often feel obliged to give concerning the care of the mouth, will surely carry with it more force after such an examination. It is not necessary that our regular business hours should be taken up in this way, or *all* of our leisure time, but the wise use of the spare moments, the half hour before or after office hours, when well occupied, will tell wonderfully in the aggregate. We will be surprised ourselves at the results which follow a systematic use of time which is often idled away. There are other subjects of equal importance with microscopy; such are entymology and histology, etc. To be well grounded in our profession, we need to go back to the *beginning*, to study life histories from the cell, then we may go on to higher developments, feeling sure our course is the right one. How much more satisfactory will be the knowledge thus gained than that obtained by reading the results of the labor of others. Then, too, the variety and diversion from daily duties will form a balance wheel. There will be food for thought, and material for conversation. It will not be necessary to "talk shop" quite so often, perhaps. There is no better way to obtain the much coveted recognition by the medical profession than to be able to meet them half way, and show a worthiness to an equal footing.

If the knowledge thus gained were of use to us only in our professional duties, it would be sufficient, but there are other advantages. A person with a mind well informed, with all the faculties well developed, must have an influence in the social circle in which he moves. Culture will tell. Our own self-respect is increased, and this we must have if we would be respected by others.

"A man is no better than the company he keeps," is a true saying. It is equally true of our mental society—the books we read. We are unconsciously elevated by reading the thoughts of wise men; we have the feeling of being in good company.

We had not thought necessary to try to prove the necessity for constant application, if we would keep pace with the times. The sluggish pool becomes muddy, while the clear sparkling stream is the one that *moves*.

All scientific men recognize the necessity for constant study, even after they have done with school and teacher. New theories are constantly being advanced, new discoveries being made, with which they are obliged to be familiar. And what an incentive to action is the thought that the origin of these theories and discoveries is confined to no rank or station. You and I have an equal chance with the rest. A dentist was heard to say, after receiving the first volume of the "American System of Dentistry," that it would do him little good, because he already had it all in his head! (Unfortunately, few of us are blessed with such retentive heads.) The poor man thought, or wanted others to think, that he had no more need of study. It is the wise who "dare to be ignorant of many things." Most of us must be willing to work if we would win.

With all these plans and suggestions for systematic use of spare time, we would not forget that very excellent adage about "all work and no play." And yet, by forethought, even our recreation may take such form as to be more beneficial and satisfactory than if taken "as it happens." To be *recreation* at all, one's individual tastes should be consulted. One may have a natural taste for entymology, and especially enjoy prying into nature's secrets as she holds them in the form of the tiny insect. Then, too, the subject may have a closer relation to the higher orders of life than we would at first suppose. For instance, in the pursuit of our calling, we have thought of the teeth as organs belonging exclusively to the oral cavity. How interesting then, when, on examination of some of the lower orders, to find these useful organs *in the stomach.* We then realize that there *might* be a worse locality for a cavity than the posterior side of a third molar. Thus we have gained a point in comparative anatomy, as well as a cause for thankfulness that our duties do not lie among that order of beings. While we may thus be carried away with the beauties of animal life, another may be equally fascinated by the classification of plants which many of us pass by unheeded every day. The little knowledge of botany we gained in our school days may thus, for the first time, be put to a pleasurable use.

Or the inclination may be toward a more solid subject, then roam the fields and geologize, if the taste leads in that direction. If the habit of late hours, formed during college life, is hard to break up, astronomy offers a grand opportunity for employment of such time, especially if one is so fortunate as to have a congenial companion, who will help him to hold up the gate while gazing into the heavens, and be a willing listener while you discuss the subject of *double stars.*

To some a good horse will be just what is needed for exercise, and a better companion any time than poor society in a close room, filled with the fumes of tobacco; or the less troublesome "silent steed," the bicycle, may sometimes be well substituted. If neither of these strike the fancy, perhaps that other venerable animal, long known by the name of Pegasus, may occasionally come, with mute appeal for a canter. Yield to the impulse then, and write. Write for *fun,* write because you must (but don't read it to your friends, unless you have some very indulgent ones), and if you be moved to send something to some journal for publication, don't throw down your pen when it is "declined with thanks," but keep on writing whenever the mood comes upon you. It will prove a good escape valve for many an inward explosion. If some unforeseen occurrence sets the blood boiling, and, for the sake of your reputation as a gentleman you *must* hold your tongue, go home and write it all up in your best style. Give vent to unutterable sentiments, if you must, it will harm no one, and you will feel better. Then forget it as soon as possible. Or, if you think you have made a discovery which will do good to some one else in the profession, or have been successful in overcoming some difficulties, be generous, and let the rest of us know about it by sending an article on the subject to some of our publications. If we *could* have the courage to confess some of our *failures* occasionally, it might prove edifying to some one else; at least it would be encouraging to know that others fail sometimes.

To us who are confined in an office all day breathing not the purest atmosphere, and a great deal of the time in a cramped unnatural position, some out-door exercise, with pure air *every day*, is an absolute necessity. If the mind is pleasantly occupied in some such way, as before suggested, we will not pay much attention to the manner of locomotion. A pleasant companion will make a long walk seem short. We will walk miles in search of a rare plant, or geological specimen, or run almost as far to secure a beautiful butterfly. So, if we cannot afford horse or bicycle, our recreation and exercise may be taken without them and be made a pleasure, with pleasant thoughts for company. Perhaps as good a way as any to make sure of the daily exercise, is to have house and office some distance apart, for no one is averse to walking when under spur of a good appetite. Of course there are always books to be read, the papers and magazines, but these should not be exclusively for information or instruction. A good, healthy novel, occasionally, is very restful, while books of travel are next best to taking a trip oneself, and a good deal cheaper.

Then we have social responsibilities. It was said in the beginning: "It is not good for man to be alone." We need society, and if we would grow symmetrical, we must brush against others, have the angles rounded by moving among people, our conversational powers strengthened by exercise. In this day of literary clubs and reading circles, the best advantages are offered for meeting the best people, and it must not take any one long to find his level and have his intellectual measure taken.

There is another point which I would like to mention, one upon which I fear we sometimes forget our dignity, and that is *professional* etiquette. It is not wise to try "to build ourselves up by pulling others down." What if our fellow practitioner across the street *is* an old quack, and we are positively assured of the fact, it will do no good to tell our patients so. We will gain the respect of others, and retain our own self-respect, too, by keeping still. A wise divine has said, "keep clear of personalties in conversation." This is a good rule for every one. A well informed mind will find plenty of innocent topics of conversation without indulging in such questionable occupation as talking about one's neighbors. If we really deserve it, we need not fear but that it will be found out how much superior we are to all the other dentists in the place.

The time had better be spent in answering such questions as "What makes a tooth ache after the nerve is dead?" "Doesn't it injure the enamel to have the teeth cleaned?" etc. These and much less sensible questions *are* asked, and must be answered as minutely and pleasantly as if it was the first time the question had ever been asked. A vast amount of patient politeness, or polite patience, is required, if we would have friends. Without regard to social position, all must receive a due portion of attention. There is no formula for these things; it all lies in a nutshell. Be a lady, be a gentleman, at all times and in all places, then this gentleness of manner, this thoughtfulness for others, will be spontaneous, and need not be studied. I can cite you to no better example of this true gentility than the "man of sorrows," of whom it is said "he pleased not himself."

How often we hear the remark, "Doctor so and so is an excellent dentist, but he is *so rough*." And some of us do sometimes forget that *roughness* is not another name for *skill*. It is in gentleness and deftness of hand where my own sex may excel, if at all, not saying, however, that these desirable qualities are confined to woman, but we seem to have the credit among suffering humanity of possessing them in a higher degree than our brothers. This is one of our strongholds, ladies, and let us prove ourselves worthy of it. We may often feel thankful that we were born women, and thankful, too, that we have chosen this profession, when some pale, delicate sister, or shrinking child, comes to *us* for relief on the strength of our reputed sympathy and delicacy of touch. Heretofore I have used the masculine form of the personal pronoun only because of common usage, and not that both sexes were not in mind, for what applies to one applies to the other in study, in wise use of time, and in recreation, as well as in our duties to society and to our profession. These are laws which know no difference in man and woman, and the penalty attached for violation is, in all cases, the same. The laws of health are not less stringent than the moral law. The dentist, if any one, has need of all possible control of nerve and muscle, and this can come only from the best physical condition. A person who is the victim of dyspepsia, one of our most common ailments, has little self-control, and cannot hope to control or soothe a nervous patient, to say nothing of one's own enjoyment.

> "Nor love, nor honor, wealth nor power,
> Can give the heart a cheerful hour
> When health is lost. Be truly wise,
> With health all taste of pleasure flies."

If our work were merely to carve out cavities in blocks of ivory and fill them up with gold, the mental strain would be nothing, but we are to deal with living tissues, whose delicate structure responds to our every touch. It is consciousness of this, this giving out of human sympathy, which tells upon our sympathetic organism, until we feel that a portion of our very life was constantly being drawn away from us. This brings a weariness to which manual labor is an entire stranger, and which is overcome only by complete change of occupation of brain and hand in a way to make us forget all our work and worry. The antidote may be summed up in this short sentence: "Eat well; sleep well; take plenty of exercise."

Could these rules be faithfully observed, what pictures of perfect humanity would fill these seats at the next semi-centennial anniversary, when we older ones, who have not had the benefit of such timely advice, have been called to seats "higher up," leaving room for those of whom may it be said: "Mark the perfect man, and behold the upright." Then will the "U. of M." have a corps of alumni of whom she will have reason to feel as proud as does the heart of England's Queen in this, *her* day of jubilee, over subjects not more loyal or loving. Then will there have been many changes; crooked paths will have been made straight; theories, which may now seem like fancies of a bewildered brain, will have been verified. Appliances for facilitating operations will have been invented; sure pain obtunders and local anæsthetics will have been discovered, thus making the dental office a haven of rest, and a safe refuge from the darts of pain and disease. With prophetic vision we look ahead, and the revelation almost starts a feeling of envy of the future dentists, blessed with so many improvements.

And yet we would not look lightly upon the pleasures of the passing hour, for surely this is a "red letter day" to us all, when we see such coming together of youth and beauty, wit and wisdom, as makes us wish to stop the flight of our own years for a time, and fills us with a spirit kindred to that which inspired Macaulay when he wrote:

> "God send Rome one such other sight,
> And send me there to see."

